# Effectiveness of a Comprehensive Stress Management Program to Reduce Work-Related Stress in a Medium-Sized Enterprise

**DOI:** 10.1186/2052-4374-26-4

**Published:** 2014-02-13

**Authors:** Shin-Ae Kim, Chunhui Suh, Mi-Hee Park, Kunhyung Kim, Chae-Kwan Lee, Byung-Chul Son, Jeong-Ho Kim, Jong-Tae Lee, Kuck-Hyun Woo, Kabsoon Kang, Hyunjin Jung

**Affiliations:** 1 Department of Occupational and Environmental Medicine & Institute of Environmental and Occupational Medicine, Busan Paik Hospital, Inje University, 75, Bokji-ro, Busanjin-gu, Busan 633-165, Republic of Korea; 2 Occupational Medical Examination Center, Good Morning Hospital, 110, Samsan-ro, Nam-gu, Ulsan 680-804, Republic of Korea; 3 Department of Occupational and Environmental Medicine, Soonchunhyang University Gumi Hospital, 179, Gongdan 1-dong, Gumi-si, Gyeongbuk 730-706, Republic of Korea; 4 Education & Future Center, Hyunjin Materials. Co., Ltd, 1201-4, Jisa-dogn, Kangseo-gu, Busan 618-230, Republic of Korea

**Keywords:** Health promotion, Stress/psychological, Workplace, Intervention studies, Participatory action-oriented training

## Abstract

**Objectives:**

To assess the effectiveness of a comprehensive workplace stress management program consisting of participatory action-oriented training (PAOT) and individual management.

**Methods:**

A comprehensive workplace stress management program was conducted in a medium-sized enterprise. The baseline survey was conducted in September 2011, using the Korean Occupational Stress Scale (KOSS) and Worker’s Stress Response Inventory (WSRI). After implementing both organizational and individual level interventions, the follow up evaluation was conducted in November 2011.

**Results:**

Most of the workers participated in the organizational level PAOT and made Team-based improvement plans. Based on the stress survey, 24 workers were interviewed by a researcher. After the organizational and individual level interventions, there was a reduction of several adverse psychosocial factors and stress responses. In the case of blue-collar workers, psychosocial factors such as the physical environment, job demands, organizational system, lack of rewards, and occupational climate were significantly improved; in the case of white-collar workers, the occupational climate was improved.

**Conclusions:**

In light of these results, we concluded that the comprehensive stress management program was effective in reducing work-related stress in a short-term period. A persistent long-term follow up is necessary to determine whether the observed effects are maintained over time. Both team-based improvement activities and individual interviews have to be sustainable and complementary to each other under the long-term plan.

## Introduction

Work-related stress can be defined as the harmful physical and emotional responses that occur when the requirements of the job do not match the capabilities, resources, or needs of the worker [1]. In the case of individual workers, work-relate distress can cause job dissatisfaction, absenteeism, accidents, voluntary unemployment, and finally lead to a decreased quality of life [2]. For employers, work-related stress has a significant effect on the risk of worker’s being injured in an occupational accident, which increases costs and lost productivity, and finally leads to a deterioration of management [3]. From the perspective of the nation and the community, these results have negative impacts such as competitive decline [4]. All workers can suffer from work-related stress, which has a huge social cost. Thus, work-related stress is not only an individual risk factor, but also a risk factor for employers, the community, and the country’s development.

In 27 countries of the European Union (EU) in 2005, 22.3% of the workers had symptoms caused by stress [5]. In the United Kingdom, estimates of work-related stress was 428,000 cases (40%) out of all work-related illnesses. This could be a factor responsible for the high number of lost work days, which reached 10.4 million in 2011/12 based on the Labour Force Survey data [6]. In a study conducted in Korea, 346 workers (22%) among 6,977 experienced critical levels of stress [7]; this was widely reported, particularly in small and mediumsized enterprises. Male white-collar workers with high job stress suffered from more depression, anxiety, and stress symptoms than workers with low job stress in an automobile company [8]. Depressive symptoms of male workers had a greater impact on work-related stress than the quality of sleep and fatigue in the case of a small manufacturing company [9]. The self-perceived fatigue of white-collar male workers had a greater impact on work-related stress than the psychosocial factors in the case of small and mediumsized enterprises [10].

In order to manage work-related stress, effective interventions applicable to the workplace are required. Internationally, various stress interventions in the workplace have been conducted on the basis of work-related stress research. Depending on whether the intervention focuses on an individual’s psychological resources (individual level) or occupational context (organizational level), there are two different approaches to managing work-related stress. At the individual level, relaxation and, cognitive-behavioral techniques have been applied to improve an individual’s psychological resources and responses. At the organizational level, job adjustment and workplace communication activation have been applied to improve the occupational context. In previous intervention studies at only the organization level, there was little impact on individual variables. Individual level interventions had more influence on psychosocial, physiological, and organisational aspects [11]. In a meta-analysis of workplace stress interventions, the cognitive-behavioral approach was found to be the most effective technique [12,13]. One year after the individual intervention, biofeedback and muscle relaxation showed reduced absenteeism. In the following year, however, the effects of the intervention disappeared and the variables returned to their previous status [14]. In a similar study, individual level interventions showed only short-term effects [15]. Secondary or tertiary prevention, which was usually applied to the individual level intervention, strengthened coping with stress that had already occurred, but primary prevention, which was usually applied as an organizational level intervention, reduced the cause of stress. Therefore, only secondary or tertiary prevention without primary prevention seemed to have a limited effect on the reduction of work-related stress. In previous reports, both individual and organizational management for improving workplace mental health were important to manage the causes of stress in the workplace [12]. The improvement of the psychosocial work environment is helpful to the improvement of the mental health of workers [16], and mental health is closely related to productivity improvement [17]. If intervention measures are not mutually exclusive but complementary, an even greater effect can be observed. Therefore, comprehensive stress management should be emphasized [18].

Participation in workplace health promotion has increased significantly [19,20]. Involvement of the participants can enhance autonomy, justice, and social support, which are the basic components of work-related stress [21]. Participatory action-oriented training (PAOT) is one of the methods for engaging employees in workplace interventions. Workers who participate in this workshop discover the problems caused by their own work environment. The program suggests appropriate solutions to workplace problems and improves the work environment [20]. This participatory approach to reducing work-related stress in the work environment reduces psychosocial stressors. It also reinforces the buffer elements against stress [22]. The application of both participatory approach methods and psychosocial stress reduction methods has been effective in improving a worker’s mental health and productivity [23].

In this study, a comprehensive stress management program was applied to a medium-sized enterprise that experienced an increase in work-related stress due to rapid growth. The comprehensive stress management program was composed of both participatory organizational intervention for improving the work environment and individual interventions for reinforcing the coping skills against stress. Further, we implemented the stress management program and evaluated its effects.

## Materials and methods

### Setting and subjects

**Figure 1:**
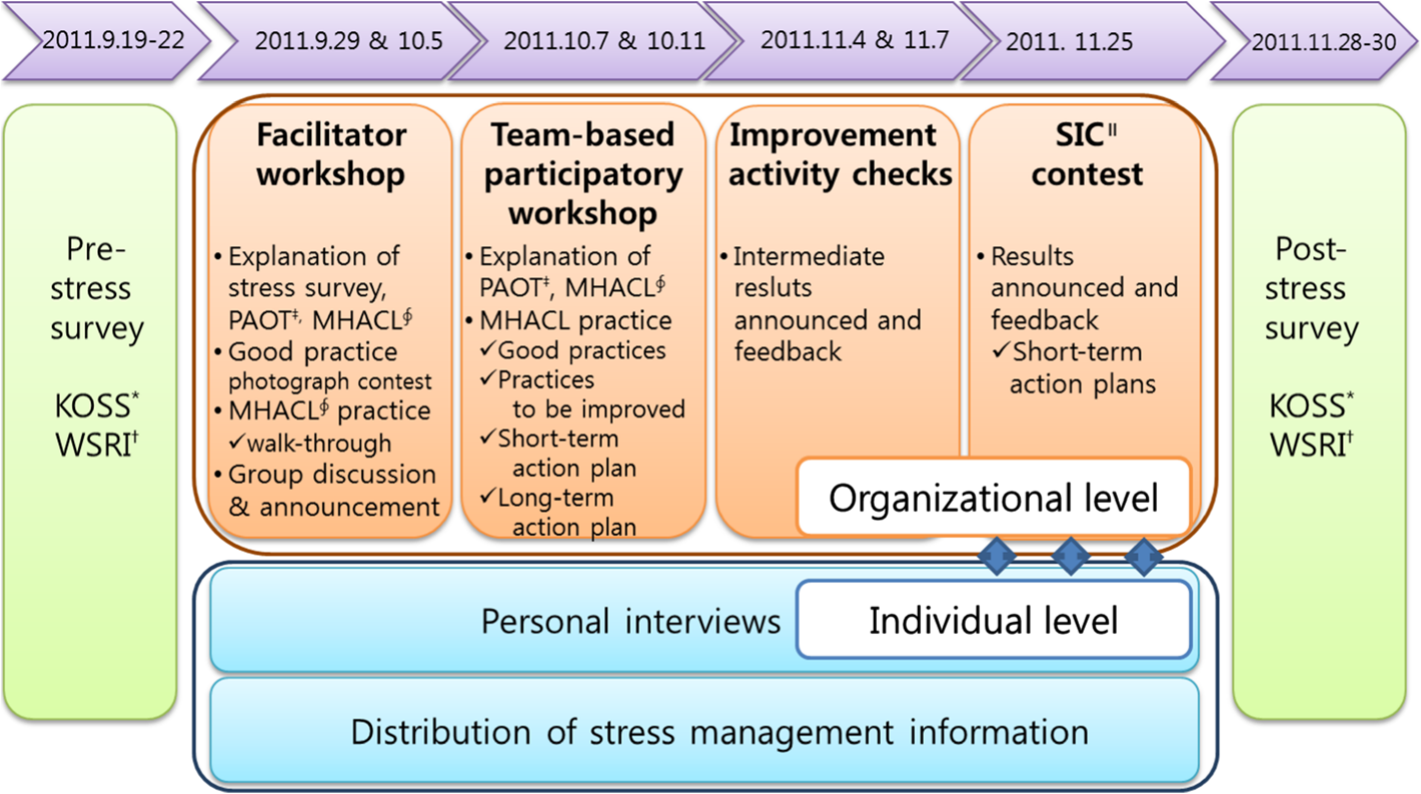
**Outline of comprehensive stress management program.** *Korean Occupational Stress Scale, ^†^Worker’s Stress Response Inventory, ^‡^Participatory action-oriented training, ^∮^Mental Health Action Checklist ∥Simple, inexpensive, clever.

This study was conducted in a mediumsized metal forging company that manufactures large parts for marine engines, wind power components, and crank engines. From 2005 to 2011, the company’s annual revenue increased up to 70.4% and the number of employeesincreased by three times. However, problems occurred with the rapid growth of the scale of the company and changes in the organizational structure. Since 2007, to solve this problem, the company has run a variety of projects. An internal evaluation revealed that work-related stress was high due to a change in the organizational culture. Therefore, a workplace stress management program was conducted from September 5 to November 25, 2011. This company had 19 teams of white-collar workers and 12 teams of blue-collar workers, and the total number of employees was 295. The white-collar workers engaged in quality assurance, management support, domestic sales, international sales, production management, technology research, financial strategy, integrated purchasing and trade, quality management, and financial planning. The blue-collar workers engaged in pressing, heat treatment, support, cutting, and maintenance. Seven teams were excluded due to distance of another location or the continuous operation of their machinery. The participants were 123 white-collar workers comprising 13 teams and 129 blue-collar workers comprising 11 teams. Four workers could not respond to the pre-stress survey for reasons such as sick leave and external training. During the study period, 13 workers retired. Five workers could not respond to the post-stress survey for reasons such as sick leave and vacation. There were anonymous responses by 8 workers in the pre- and 16 workers in the post-stress survey; therefore, we could not perform the paired analysis for them. Finally, 91 white-collar workers and 120 blue-collar workers were analyzed in the paired analysis. An outline of the comprehensive stress management program is illustrated in Figure 1.

### Stress survey

All of the workers were asked to fill out self-reported questionnaires on September 19–22 and November 28–30, 2011. The same questionnaires were administered in the pre- and post-stress surveys. The questionnaires included general questions related to gender, age, educational level, and position. To compare the before- and after-program effects, we used the Korean Occupational Stress Scale (KOSS) and Worker’s Stress Response Inventory (WSRI). The questionnaires were distributed and collected in sealed envelopes by the company’s human resources department. The collected questionnaires were submitted to the researchers for analysis. The participants were clearly told that their responses would be kept confidential from the company before we carried out the survey as described above.

The KOSS was used to evaluate work-related stressors to reflect the domestic work environment. The KOSS includes eight subscales with 43 items, including the physical environment, job demands, insufficient job control, interpersonal conflicts, job insecurity, organizational system, lack of rewards, and organizational climate [24]. The WSRI was used to measure stress-related symptoms associated with the job [25]. The WSRI was a combination of two tools. One was a short-form questionnaire based on the stress response scale developed by Koh [26], and the other was derived as a subscale of the Korean Version of the Occupational Stress Inventory (K-OSI). WSRI included four subscales with 26 items, namely depressive symptoms, physical symptoms, anger symptoms, and work-related symptoms.

### Intervention

#### Organizational level

The PAOT was performed using a mental health action checklist focused on improving the work environment [27] from September 29 to November 25, 2011. The intervention attempted to solve the problems on a team-based approach. Improving the workplace environment was focused on the reduction of work-related stress through the participation of workers. The PAOT emphasized a voluntary initiative of the workers rather than the traditional rule-based approach [20]. Facilitator workshops were performed first, followed by team-based participatory workshops. One month after the workshops, improved activity checks were conducted by the stress management team. Finally, a simple, inexpensive, clever (SIC) contest was held for determining the recipients of the achievement awards, and the improved activities were announced.

##### Stress management team

The stress management team was involved in the overall program planning, implementation, follow up, and evaluation. The team consisted of occupational and environmental medicine (OEM) specialists, residents, and occupational health nurses. Two OEM specialists who had been trained on PAOT educated one specialist, two residents, and nine occupational health nurses. The stress management team selected the Mental Health Action Checklist (MHACL) [27] to evaluate workplace stress voluntarily and to facilitate the improvement brought about by the workers. Meetings with the human resources department were held to discuss the workplace situation and to encourage workers to participate in the stress management program in the preparation phase.

##### The MHACL focused on the improvement of the work environment

This study used the Korean version [27] of the Japanese MHACL [28], which was translated by Park to fit domestic conditions. The MHACL is a useful guide for work-related stress assessment and provides the available options of feasible improvement in various sectors. MHACL consists of 24 good practices in six areas: (1) participation in work planning, (2) working time and organization, (3) ergonomic work methods, (4) workplace environment, (5) mutual support in the workplace, and (6) preparedness and care [28]. The checklist was developed to facilitate improvement activities. Participants can discuss work-related stress or the work environment effectively using the MHACL, and find ways to improve their work environment followed by reducing work-related stress.

##### PAOT workshop implementation

Facilitator workshop

The facilitator workshop targeted 24 team leaders and was conducted twice, once on September 29 and next on October 5, at the company’s auditorium. The first workshop lasted 2 hours. The stress management team explained the stress survey results and introduced the PAOT concepts and the MHACL. The second workshop lasted 4 hours and was focused on the interactive learning of how to run a PAOT in detail and a good practice photograph contest. The good practice photographs collected in previous PAOT workshops at other workplaces were exhibited, and each participant selected three pictures that were thought to fit their workplace well. It enabled participants to learn good practices by knowing the reason for their selection, helped them to understand the MHACL on the basis of the good practices, and motivated them to improve their work environment. After the photograph contest, the participants created their own MHACL from the original MHACL by selecting items that fit their work environment (checklist redesign). The participants conducted a walk-through of their workplace to practice the redesigned MHACL. The “good practices” and “practices to be improved” were discussed in the group meetings and presentations.

Team-based participatory workshop

The team-based participatory workshop was conducted with 86 white-collar workers in 13 teams and 108 blue-collar workers in 11 teams. The workshops lasted 2 hours for each team and were conducted on October 7, 10, and 11 in 2011. The stress management team explained the concepts of work-related stress and mental health in the workplace, and introduced PAOT concepts and MHACL. The participants of each team discussed the items of the MHACL and selected three good practices and three practices to be improved. Short- and long-term action plans for the practices to be improved were developed. The facilitators participated in the discussion of each team, encouraged workers to make an active commitment, and if necessary, answered and provided advice on the questions. At the end of the workshop, each team announced the results of the discussion: good practices, practices to be improved, and short-term and long-term action plans.

Improvement activity checks

The researchers summarized the results of the team-based participatory workshop. The human resources department circulated the document of action plans in each team for the purpose of the reconfirmation and stimulation improvement activity on October 31, 2011. The intermediate improvement activities were collected until November 4, 2011. The researchers monitored the implementation of the action plans. If the action plans did not run appropriately, the researchers investigated the problems and provided relevant advice.

Simple, inexpensive, clever (SIC) contest

The SIC contest was held with team leaders who participated in the facilitator workshop. The implementation of action plans was evaluated. Team leaders presented their improvement activities. Good practices to reduce work-related stress were shared. The most simple, inexpensive, and clever improvement practices were chosen by a vote and awarded.

#### Individual level

##### Personal interviews

The interview subjects were selected: 9 workers above 31 points on the WSRI and 9 workers above 22 points on the sleep questionnaire. Among them, 15 workers were interviewed; of the 3 not considered, 1 had retired and 2 were redundant. Finally, 24 workers were interviewed, including 9 voluntary applicants who wanted to be interviewed. The interviews were conducted five times every Tuesday afternoon from October 18 to November 22, 2011.

The occupational and environmental medicine (OEM) specialist interviewed subjects in a separate room. Brief descriptions of subjective stressors, responses, and coping methods for work-related stress were provided on paper before the interview. The coping part of the K-OSI [29] composed of recreational activities, self-care, social support, and rational and cognitive coping was also completed, and the OEM specialist evaluated the coping skills of individual workers. The interview began with the individual results of the KOSS and WSRI. The OEM specialist encouraged the workers to speak freely about the stress caused by their work. Based on these data, the OEM specialist helped workers to identify their work-related stressors and strengthen their coping skills.

##### Disseminating stress management information

Information about work-related stressors and coping skills were distributed seven times through the company’s computer network, every Tuesday from October 11 to November 22, 2011. This information included details regarding the following: identification of the stressors, goal setting for stress management, cognitive-behavioral therapy, anger management training, how to induce sleep, abdominal breathing techniques, and muscle relaxation techniques.

### Statistical analysis

A chi-squared test was used to analyze general characteristics and work-related characteristics. The descriptive statistics were expressed in terms of frequency and percentage. In order to confirm the effect of the comprehensive stress management programs, a paired t-test was used to compare the pre- and the post-stress surveys, which included the KOSS and WSRI. Reliability was determined by the value of Cronbach’s alpha. Statistical analyses were conducted using SPSS 18, and the significance level was fixed at 0.05.

## Results

### General characteristics

A total of 211 workers were eligible for the study. There were 120 (56.9%) blue-collar and 91 (43.1%) white-collar workers. The blue-collar worker group was composed only of male workers, and the white-collar worker group had 73 (80.2%) male workers. Most of the blue-collar and white-collar workers considered in this study were in their thirties. With respect to the education level, the group of “high school or less” had 70 blue-collar workers (58.8%) and the group of “college graduates” had 49 blue-collar workers (41.2%). The group of white-collar workers was composed mostly of college graduates, 89 (98.9%). With respect to the employment status, 72 blue-collar workers (60.0%) belonged to the “general” group. In contrast, 46 white-collar workers (51.1%) belonged to the “general” group, in which the general and supervisor’s rate was relatively evenly distributed (Table 1).

**Table 1:** General characteristics of blue-collar workers and white-collar workers

	**Blue-collar workers**		**White-collar workers**		
	**No.**	**%**	**No.**	**%**	p-value*
Gender					
Men	120	100.0	73	80.2	<0.001
Women	0	0.0	18	19.8	
Age					
20-29	10	8.4	29	32.6	<0.001
30-39	80	67.2	52	58.4	
40-49	24	20.2	6	6.7	
50-59	5	4.2	2	2.2	
Educational level					
≤High school	70	58.8	1	1.1	<0.001
≥College	49	41.2	89	98.9	
Employment status					
General^†^	72	60.0	46	51.1	<0.001
Supervisor^‡^	48	40.0	44	48.9	

*p-value by chi-squared test.

^†^“general” means mere clerk.

^‡^“supervisor” means higher position than mere clerk such as section chief or director.

### Action plans

The three action plans that were mentioned the most were as follows: First, in the case of blue-collar workers, the short-term action plans were to wear safety protective equipment and respiratory protective masks, to clean the workplace, and to adjust holidays considering the work and individual schedule. The long-term action plans were to improve ventilation, lighting, and noise; to allocate flexible work hours considering the personal needs of workers; and to increase the blue-collar workforce. Second, in the case of white-collar workers, the short-term action plans were to change and clean the lighting, to hold a brief meeting before work, and to change the atmosphere of team dinners such as conversing without alcohol or watching a movie, baseball game, etc. The long-term action plans were to improve ventilation, lighting, and noise; to provide hygienic toilets and resting facilities; and to organize informal social gatherings and recreational activities (Table 2).

**Table 2:** Action plans presented at the workshop

	**Short-term action plans**	**Long-term action plans**
Blue-collar Workers	1. To wear personal protective equipment	1. To improve ventilation, lighting, and noise
	2. To clean the workplace	2. To allocate flexible work hours considering personal needs
	3. To adjust holidays considering the work and individual schedule	3. To increase the workforce
White-collar Workers	1. To change and clean the lighting	1. To improve ventilation, lighting, and noise
	2. To hold a brief meeting before work	2. To provide hygienic toilets and resting facilities
	3. To change the atmosphere of the team dinner	3. To organize informal social gatherings and recreational activities

### Comparison of the KOSS score before and after the program

Table 3 shows the changes in the mean subscale scores of the KOSS obtained through the pre- and post-stress surveys. The blue-collar workers exhibited a statistically significant intervention effect in the case of physical environment (p = 0.003), job demands (p = 0.001), organizational system (p = 0.001), lack of rewards (p = 0.035), organizational climate (p = 0.048), and total score (p < 0.001). The white-collar workers exhibited a statistically significant intervention effect in the case of the organizational climate (p = 0.011). Although not statistically significant, the subscales of insufficient job control, interpersonal conflicts, and job insecurity were improved in the case of blue-collar workers. Although not statistically significant, the subscales of job demands, insufficient job control, interpersonal conflicts, organizational system, lack of rewards, and total score were improved in the case of white-collar workers (Table 3). The value of Cronbach’s alpha was in the range of 0.706 to 0.880.

**Table 3:** Comparison of the KOSS scores obtained through the pre- and post-stress surveys

	**Blue-collar workers**								**White-collar workers**							
		**Pre-**			**Post-**					**Pre-**			**Post-**			
**Subscales**	**N**	**Mean**	**SD**	**CB α**	**Mean**	**SD**	**CB α**	P^*^	**N**	**Mean**	**SD**	**CB α**	**Mean**	**SD**	**CB α**	P^*^
Physical environment	117	65.24	19.16	0.794	59.45	20.29	0.818	0.003	89	36.44	20.11	0.884	36.45	18.50	0.777	0.995
Job demands	119	45.83	15.47	0.766	40.86	14.18	0.816	0.001	89	55.20	17.96	0.874	53.84	14.57	0.760	0.396
Insufficient job control	119	54.51	13.71	0.797	52.16	12.32	0.830	0.054	89	52.44	15.48	0.874	49.81	19.02	0.788	0.248
Interpersonal conflicts	119	38.10	15.86	0.786	36.98	14.13	0.811	0.498	89	39.32	17.73	0.863	35.96	14.84	0.747	0.077
Job insecurity	116	46.24	11.03	0.795	45.74	12.51	0.828	0.649	88	43.56	16.60	0.880	43.88	12.58	0.778	0.838
Organizational system	119	57.98	17.94	0.750	53.06	16.26	0.797	0.001	90	53.12	17.44	0.856	50.64	14.46	0.720	0.162
Lack of rewards	120	52.36	14.43	0.767	49.51	14.19	0.795	0.035	90	50.43	17.29	0.858	48.65	13.90	0.740	0.265
Organizational climate	118	38.70	16.03	0.757	36.02	15.29	0.799	0.048	90	47.55	17.35	0.856	42.71	17.21	0.720	0.011
Total score	113	49.82	9.20	0.734	46.39	9.45	0.776	<0.001	88	47.26	12.23	0.844	45.39	9.03	0.706	0.112

*p-value by paired t-test.

KOSS: Korean Occupational Stress Scale, SD: Standard deviation, CB α: Cronbach’s alpha.

### Comparison of the WSRI score before and after the program

Table 4 shows the changes in the mean subscale scores of the WSRI obtained through the pre- and post-stress surveys. Although not statistically significant, the subscales of somatic symptoms, depressive symptoms, work-related symptoms, and total score were improved in the case of both blue- and white-collar workers. The value of Cronbach’s alpha was in the range of 0.747 to 0.877.

**Table 4:** Comparison of WSRI scores obtained through the pre- and post-stress surveys

	**Blue-collar workers**								**White-collar workers**							
		**Pre-**			**Post-**					**Pre-**			**Post-**			
**Subscales**	**N**	**Mean**	**SD**	**CB α**	**Mean**	**SD**	**CB α**	P^*^	**N**	**Mean**	**SD**	**CB α**	**Mean**	**SD**	**CB α**	P^*^
Somatic symptoms	119	6.23	6.32	0.753	5.60	4.98	0.753	0.201	88	12.20	8.05	0.756	10.69	6.95	0.747	0.058
Depressive symptoms	120	4.58	5.56	0.748	3.97	4.52	0.758	0.177	89	8.96	7.10	0.752	8.16	6.20	0.747	0.247
Anger symptoms	120	3.08	3.73	0.788	3.17	3.35	0.798	0.762	89	6.68	5.22	0.791	6.70	4.26	0.791	0.972
Work-related symptoms	120	1.96	2.32	0.820	1.70	1.85	0.838	0.140	89	3.79	2.83	0.830	3.51	2.59	0.826	0.365
Total score	119	15.87	15.48	0.829	14.47	13.25	0.877	0.233	88	31.60	20.50	0.854	29.17	17.22	0.832	0.205

*p-value by paired t-test.

WSRI: Worker’s Stress Response Inventory, SD: Standard deviation, CB α: Cronbach’s alpha.

## Discussion

The comprehensive stress management program, which was composed of the organizational and the individual level intervention, was conducted to reduce work-related stress in a medium-sized enterprise. Work-related stress management has usually been focused on individual management corresponding to secondary and tertiary prevention [12]. Thus, the occupational context including physical, environmental factors that caused stress tended to be overlooked. By changing the demands of the work environment and emphasizing organizational support and responsibility, the comprehensive stress management which encompasses primary, secondary and tertiary prevention is emerging as important [18,30-32].

To reduce work-related stress, workers carried out a risk assessment of their workplace and created ready-to-run action plans by participating in a PAOT. The PAOT using the MHACL has shown a reduction in work-related stress in previous studies, similar to our findings. In a manufacturing company, female white-collar workers showed improvement in job skills, mutual support, and psychological stress [33]. In an electronic components factory, blue-collar workers exhibited an improvement in mental health and job skills [34].

The MHACL helped the participants to efficiently discuss issues related to the reduction of work-related stress through improvements in the work environment. The MHACL encompasses a variety of areas from the physical to psychosocial work environment; therefore, it can be applied to various types of workplaces [28]. In this study, the MHACL was applied to establish and implement action plans in each team. Based on the practices to be improved as determined through a discussion, each team prepared three short-term and long-term action plans. In previous studies, the MHACL was useful guidance for work-related risks and facilitated workers to set immediate goals and to plan local feasible improvements activities [20,35]. In this study, the examples of improvement activities were as follows: spraying water in order to prevent dust, adjusting work hours considering individual circumstances, and arranging regular meetings to share information. Improvement plans were made to remove obstacles causing work-related stress. Plans were made to be feasible, and improvements have been made in the desirable direction.

A comparison of KOSS scores obtained through pre- and post-stress surveys was used for evaluating the intervention effect. Blue-collar workers exhibited significant effects in the case of the subscales for the physical environment, job demands, organizational system, lack of rewards, and organizational climate. Work-related stress was reduced in the process of discovering the practices to be improved, discussing action plans with team members, and implementing improvement activities. In a previous study on the development of action plans by workers for resolving problems in the workplace, desired effects were also shown through a process of recognition and intervention [36]. In order to reduce workplace stressors and improve business efficiency and productivity, an agreement among various departments with respect to preparation for unexpected work and an understanding of task progress should be reached. White-collar workers exhibited significant effects in the case of organizational climate. Although not statistically significant, the subscales of job demands, insufficient job control, interpersonal conflicts, organizational system, and lack of rewards were improved. In the case of white-collar workers, work-related stress was induced by unreasonable communication and relationship conflicts. They felt the burden of close deadlines, work interruption due to other work, increased workload, and excessive responsibility. Work-related stress was reduced after carrying out the suggested improvements. These results were similar to those of training programs on social motivation and interpersonal conflict mediation [37]. A comparison of WSRI scores obtained through the pre- and post-stress surveys was used for evaluating the intervention effect. Although not statistically significant, the subscales of somatic symptoms, depressive symptoms, and work-related symptoms were improved. In a previous workplace-based participative intervention study, there was significant reduction in sleeping problems in hospital workers. Although not statistically significant, psychological distress, which includes anxiety, depression, aggressiveness, and cognitive problems, was also reduced after an intervention, as found in our study [39].

In the case of workplace health promotion, it is essential to promote voluntary initiatives and participation of workers throughout the entire intervention process: planning, implementation, and review [20]. In various sectors, the participatory approach has been proven to be effective in workplace stress management [33,34,38,39]. Encouragement and active participation of employees in risk assessment and control could be incorporated into the management system, followed by facilitating organizational change. In our study, most of the employees participated in the organizational level intervention. During workshops, inter- and intra-departmental communication was activated, and this effect was sustained throughout the execution of the improvement activities. Good communication helped to alleviate the problems caused by work-related stressors such as interpersonal conflicts, adverse effects to the organizational system, and deterioration of the organizational climate. These achievements would be impossible without good communication and cooperation among the departments. Assuming that the entire company was one organism made of a number of departments, it was not sufficient to enhance the individual’s coping resources with a random selection for dealing with stressors that occurred in a relationship between departments or in an organizational context. Thus, primary prevention that emphasizes worker participation has attracted attention [18].

Individual level intervention included personal interviews and the dissemination of stress management information. The interviews began with a discussion of the interviewees’ own work-related stressors and stress responses, and enhanced or supported their coping strategies against stress. Before the interview, the interviewer checked the good practices and practices to be improved for the department to which the interviewee belonged, through a team-based participatory workshop. This information helped the interviewer and the interviewee to communicate efficiently about stressors and coping methods. In addition, sensitive organizational level stressors that were difficult to describe in a public workshop were revealed in the personal interview, and feedback could be provided through improvement activity checks or SIC contest.

As this study did not apply blinding, a possible Hawthorne effect could have produced the favorable results, as workers already knew they were under the intervention. Further, this study did not include a control group; therefore, the interpretation of the results is limited. However, considering the characteristics of the workplace intervention study, the control group in the same workplace was likely to be affected by the intervention group, and the intervention effect could thereby decrease. In addition, a number of previous studies have already proven the effects of work-related stress interventions; thus, the need to select a control group is relatively low. In this study, the stress surveys relied on self-reported questionnaires; hence, an information bias could have originated from the subjective tendency to remember work-related stress. The KOSS and WSRI, as domestic evaluation scales, have certain limitations as compared to international evaluation scales. However, they provide a national reference level and have proven reliability to make an accurate assessment. Our study evaluated only the short-term effects of a comprehensive stress management program; a continuous assessment is required.

Despite these limitations, this study has great significance in terms of workplace stress intervention considering that previous stress studies in the workplace were confined to epidemiological observation in Korea. The comprehensive stress management program was conducted on the basis of stress surveys. Both organizational level interventions through participatory improvement activities and individual level interventions through interviews and information dissemination were performed. This study suggests that a comprehensive stress management program can improve the mental health of workers.

## Competing interests

Authors declare that they have no competing interests.

## Authors’ contributions

SAK, CS, MHP, JTL, KHW, and HJ conceived of the study and participated its design. MHP, CS, KHW, KK, and HJ participated in the intervention and the acquisition of data. SAK, CS, KK, CKL, BCS, JHK, and JTL performed the statistical analysis and interpretation. SAK and MHP drafted and revised the manuscript. CS coordinated the study and helped to draft and revise the manuscript. All authors read and approved the final manuscript.
